# Deciphering NF-kappaB pathways in smoking-related lung carcinogenesis

**DOI:** 10.17179/excli2024-7475

**Published:** 2024-08-19

**Authors:** Riya Thapa, Ehssan Moglad, Ahsas Goyal, Asif Ahmad Bhat, Waleed Hassan Almalki, Imran Kazmi, Sami I. Alzarea, Haider Ali, Brian Gregory Oliver, Ronan MacLoughlin, Harish Dureja, Sachin Kumar Singh, Kamal Dua, Gaurav Gupta

**Affiliations:** 1 Uttaranchal Institute of Pharmaceutical Sciences, Uttaranchal University, Dehradun, India; 2 Department of Pharmaceutics, College of Pharmacy, Prince Sattam Bin Abdulaziz University, Al Kharj 11942, Saudi Arabia; 3 Institute of Pharmaceutical Research, GLA University, Mathura, U.P., India; 4 Department of Pharmacology, College of Pharmacy, Umm Al-Qura University, Makkah, Saudi Arabia; 5 Department of Biochemistry, Faculty of Science, King Abdulaziz University, 21589, Jeddah, Saudi Arabia; 6 Department of Pharmacology, College of Pharmacy, Jouf University, 72341, Sakaka, Al-Jouf, Saudi Arabia; 7 Center for Global Health Research, Saveetha Medical College, Saveetha Institute of Medical and Technical Sciences, Saveetha University, India; 8 Department of Pharmacology, Kyrgyz State Medical College, Bishkek, Kyrgyzstan; 9 Woolcock Institute of Medical Research, Macquarie University, Sydney, NSW 2137 Australia; 10 School of Life Sciences, Faculty of Science, University of Technology Sydney, Sydney, NSW 2007 Australia; 11 Research and Development, Aerogen Limited, IDA Business Park, Galway, Connacht, H91 HE94 Ireland; 12 School of Pharmacy & Biomolecular Sciences, Royal College of Surgeons in Ireland, Dublin, Leinster, D02 YN77 Ireland; 13 School of Pharmacy & Pharmaceutical Sciences, Trinity College, Dublin, Leinster, D02 PN40 Ireland; 14 Department of Pharmaceutical Sciences, Maharshi Dayanand University, Rohtak, 124001, Haryana, India; 15 School of Pharmaceutical Sciences, Lovely Professional University, Phagwara, Punjab 144411, India; 16 Faculty of Health, Australian Research Centre in Complementary and Integrative Medicine, University of Technology Sydney, Ultimo, Australia; 17 School of Medical and Life Sciences, Sunway University, Sunway City, 47500, Malaysia; 18 Discipline of Pharmacy, Graduate School of Health, University of Technology Sydney, NSW 2007, Australia; 19 Center for Research Impact & Outcome-Chitkara College of Pharmacy, Chitkara University, Punjab; 20 Center of Medical and Bio-allied Health Sciences Research, Ajman University, Ajman, United Arab Emirates

**Keywords:** smoking, lung cancer, NF-kappaB pathway, tumorigenesis, inflammation, therapeutic interventions

## Abstract

One of the main causes of death worldwide is lung cancer, which is largely caused by cigarette smoking. The crucial transcription factor NF-κB, which controls inflammatory responses and various cellular processes, is a constitutively present cytoplasmic protein strictly regulated by inhibitors like IκB proteins. Upon activation by external stimuli, it undergoes phosphorylation, translocates into the nucleus, and modulates the expression of specific genes. The incontrovertible association between pulmonary malignancy and tobacco consumption underscores and highlights a public health concern. Polycyclic aromatic hydrocarbons and nitrosamines, potent carcinogenic compounds present in the aerosol emitted from combusted tobacco, elicit profound deleterious effects upon inhalation, resulting in severe perturbation of pulmonary tissue integrity. The pathogenesis of smoking-induced lung cancer encompasses an intricate process wherein NF-κB activation plays a pivotal role, triggered by exposure to cigarette smoke through diverse signaling pathways, including those associated with oxidative stress and pro-inflammatory cytokines. Unraveling the participation of NF-κB in smoking-induced lung cancer provides pivotal insights into molecular processes, wherein intricate crosstalk between NF-κB and pathways such as MAPK and PI3K-Akt amplifies the inflammatory response, fostering an environment conducive to the formation of lung cancer. This study reviews the critical function of NF-κB in the complex molecular pathways linked to the initiation and advancement of lung carcinogenesis as well as potential treatment targets.

See also the graphical abstract(Fig. 1).

## Introduction

Lung cancer is a major cause of mortality worldwide and a major public health problem. The main cause of lung cancer, accounting for around 85 % of instances, is tobacco use, which includes using cigarettes, cigars, and pipes (de Sousa and Carvalho, 2018[46]). Non-small cell lung cancer (NSCLC) and small cell lung cancer (SCLC) are the two most prevalent kinds of lung cancer; NSCLC is more common and develops more slowly, whilst SCLC is less common but frequently grows more swiftly (Dubin and Griffin, 2020[55]). Even with significant advancements in tobacco control, lung cancer will continue to be a leading cause of death globally for a number more decades, given present smoking habits (Hirsch et al., 2017[82]). Lung cancer is predicted to have killed 1.8 million people in 2020 (18 %) (Hoy et al., 2019[87]). Air pollution, asbestos and chemical exposure at work, and secondhand smoke are additional risk factors for lung cancer (Huang et al., 2023[90]). Early lung cancer symptoms might be insignificant or mistaken for other respiratory problems, delaying detection (Abubakar et al., 2023[1]; Chang et al., 2021[31]). Lung cancer caused by smoking is still one of the biggest worldwide health concerns and because of its significant effects on general health, it is imperative to fully understand the underlying molecular pathways of this disease (Dela Cruz et al., 2011[48]; Hou et al., 2019[86]). Lung cancer caused by smoking is one of the world's most common causes of cancer-related fatalities, and most instances are associated with cigarette smoking (Lee and Kazerooni, 2022[113]). The substances found in smoke from cigarettes can damage the genetic material in the lung cells, placing you at a considerably elevated risk of getting lung cancer (Balaga et al., 2023[11]; Malyla et al., 2023[132]). Even with significant advancements in tobacco control, lung cancer will continue to be a leading cause of death globally for a number more decades, given present smoking habits.

### Global impact of lung cancer and its association with smoking

The enormous worldwide burden of lung cancer is a reflection of both the disease's variety and the ubiquitous effect of tobacco use (Nagah and Amer, 2021[142]). According to the World Health Organization (WHO), lung cancer accounts for around 2.1 million new cases and 1.8 million deaths worldwide each year, making up almost half of the cancer burden (Nagah et al., 2019[143]). Smoking is identified as the primary causative cause of lung cancer development due to its wide range of carcinogenic chemicals (Prijić and Igić, 2021[153]). About 85 % of cases of lung cancer are caused by smoking, which is the main avoidable risk factor for the illness. The regional distribution of lung cancer incidence is closely connected with variations in smoking prevalence among regions (Rivera and Wakelee, 2016[164]). The relationship between smoking and lung cancer is further shown by the fact that countries with greater tobacco use rates also tend to have higher incidences of lung cancer (Saba et al., 2017[167]). It is critical to comprehend this global context because it emphasizes the pressing need for comprehensive initiatives to reduce the incidence and death of lung cancer caused by smoking (Shreves et al., 2023[171]; Wang et al., 2021[193]).

### NF-κB's role in the pathogenesis of smoking-induced lung cancer

The NF-κB transcription factor family is necessary for several physiological processes, such as cell survival, immune responses, and proliferation (Aswini, 2009[8]). Its involvement in cancer has been well investigated; findings indicate that dysregulation of it happens in several malignancies, including lung cancer (Chiang et al., 2023[37]). Despite its context-dependent participation in cancer and potential pro- and anti-tumorigenic properties, the precise roles played by NF-κB in smoking-induced lung cancer remain an intriguing and challenging area of investigation (Conlon et al., 2020[39]; Czyżykowski et al., 2016). Cigarette smoke contains a complex mixture of over 7,000 chemicals, which causes lung tissues to go through a sequence of reactions that lead to DNA damage, chronic inflammation, and cancer (Bao et al., 2023[12]). One important chemical event that is brought on by these events is NF-κB activation. Nevertheless, further research is necessary to determine the exact pathways by which NF-κB affects the development and course of smoking-induced lung cancer (Chiang et al., 2023[37]; Conlon et al., 2020[39]). The complicated etiology of smoking-related lung cancer includes the interplay of inherited, environmental, and lifestyle factors (Jin et al., 2021[94]). A number of carcinogens, including polycyclic aromatic hydrocarbons (PAHs), nitrosamines, and heavy metals, are present in cigarette smoke. These chemicals damage DNA, induce mutations, and alter epigenetic patterns, all of which promote the growth of cancer (Bhat et al., 2024[18]; Jomova et al., 2023[97]). Cigarette smoke not only causes genotoxicity but also chronic inflammation, which creates an environment that is conducive to the growth of cancer (Li et al., 2019[119]). The recruitment of immune cells is facilitated by inflammatory mediators such as chemokines and cytokines, which intensify the inflammatory response (Lin et al., 2021[122]). An important role for NF-κB, a master regulator of inflammation, appears in this process (Ma et al., 2020[129]). Because of its adaptability in cellular functions, NF-κB is an important participant in the intricate web of processes that are set off by exposure to cigarette smoke (Qian et al., 2022[155]). Comprehending the ways in which NF-κB influences inflammation, immunological response, and cell survival in the context of smoking-induced lung cancer is essential for the creation of focused therapeutic approaches (Bhat et al., 2023[19]; Qiu et al., 2017[156]). This review attempts to critically evaluate the molecular processes that NF-κB orchestrates in the pathogenesis of smoking-induced lung cancer, address current developments in lung cancer therapy and prevention.

## NF-κB: An Overview

An essential modulator of cellular responses, NF-κB affects a wide range of physiological functions. In the realm of cancer, NF-κB is a an essential molecular conductor that directs complex signaling cascades that advance the illness (Williams and Gilmore, 2020[197]; Zhang et al., 2017[207]). When it functions properly, NF-κB is essential for immunological responses, inflammation, and cell viability (Wibisana and Okada, 2022[196]). On the other hand, smoking-related lung cancer is one type of cancer linked to abnormal NF-κB activation (Bhat et al., 2023[20]; Sun, 2011[178]). Understanding the complex involvement of NF-κB in cellular dynamics provides a crucial foundation for understanding the complexities of smoking-induced lung cancer and opens up new avenues for targeted therapeutics intended to disrupt the cascade of events orchestrated by this molecular player.

### NF-κB's involvement in cancer

In an environment of cancer, NF-κB plays a dual role as an adversary and a protector at the junction of cellular destiny (DiDonato et al., 2012[50]). As a guardian of cellular survival, NF-κB orchestrates immune responses and maintains tissue homeostasis when it is operating appropriately (Eluard et al., 2020[58]). On the other hand, NF-κB becomes a potent cancer-spread promoter when it is activated improperly (Fan et al., 2013[60]). NF-κB performs a myriad of functions in the complicated topography of cancer. It increases chronic inflammation, promotes cell proliferation, and inhibits apoptosis, all of which help to create an environment that is conducive to tumor growth (Hoesel and Schmid, 2013[84]; Khan et al., 2020[101]). When smoking causes lung cancer, the carcinogenic components of tobacco smoke become the crucial factor that triggers NF-κB activation (Bhat et al., 2024[21]; Lalle et al., 2021[111]). Lung cancer develops, advances, and is maintained by a number of molecular pathways that are triggered and maintained by this activation (Lawrence, 2011[112]).

### NF-κB activation in response to smoking-induced inflammation

**Figure 2:**
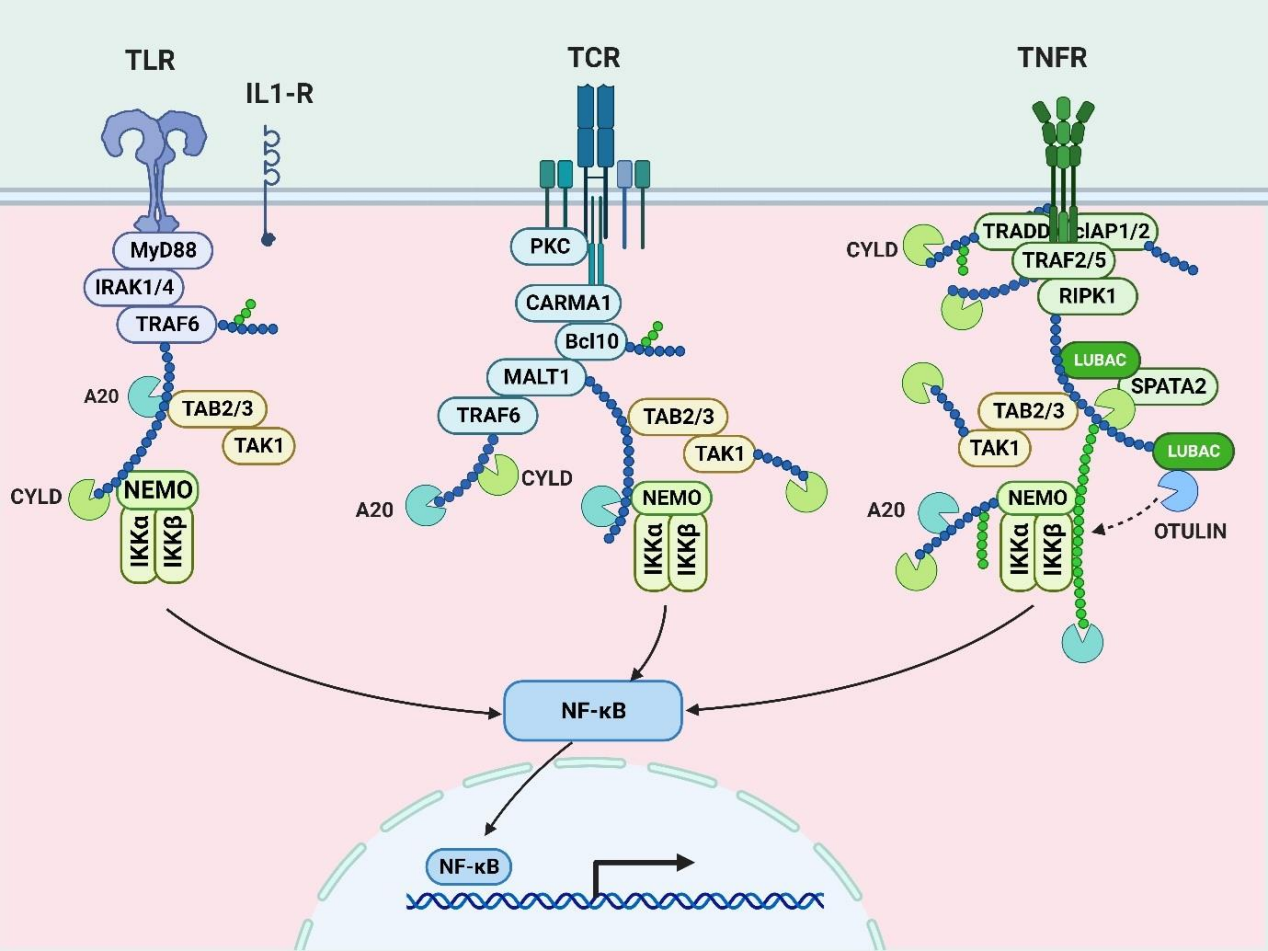
This image shows NF-κB pathways, pivotal in immune responses. Toll-like receptors (TLR), T-cell receptors (TCR), and tumor necrosis factor receptors (TNFR) activate NF-κB, influencing immune and inflammatory processes.

It is known that inhaling tobacco smoke triggers the transcription factor NF-κB, which is essential for inflammation (Conlon et al., 2020[39]). In one study, gingival epithelial cells (Ca9-22) were treated to 10 % cigarette smoke extract (CSE) for six hours (Dang et al., 2020[43]). The results showed that the RAGE was significantly more expressed, and this caused the NF-κB signaling pathways to be activated (Deng et al., 2023[49]). Via quantitative PCR and immunoblotting, the enhanced RAGE expression following CSE therapy was confirmed. Moreover, the activation of Ras and additional NF-κB signaling intermediates was observed (Gonçalves et al., 2011[68]). The previously stated activation suggests that NF-κB is necessary for the cell's response to inflammation brought on by smoking (He et al., 2020[76]). Further evidence that NF-κB is active in the inflammatory cascade comes from the study's evaluation of larger levels of pro-inflammatory cytokines, such as IL-1β and IL-6 in the cell culture media of CSE-exposed cells than in controls (Hirano, 2021[80]; Huang et al., 2022[91]). Co-treatment with CSE and SAGEs effectively decreased the inflammatory responses, indicating the involvement of NF-κB in smoking-induced inflammation. These findings demonstrate the involvement of NF-κB in the molecular pathways of inflammation caused by cigarette smoke, offering insights that might aid in the development of focused therapies for oral conditions exacerbated by tobacco smoke exposure (Kunnumakkara et al., 2020[110]; Rastrick et al., 2013[160]) (Figure 2(Fig. 2)).

## Mechanisms of NF-κB Activation in Smoking-Induced Lung Cancer

### MARCKS pathway

**Figure 3:**
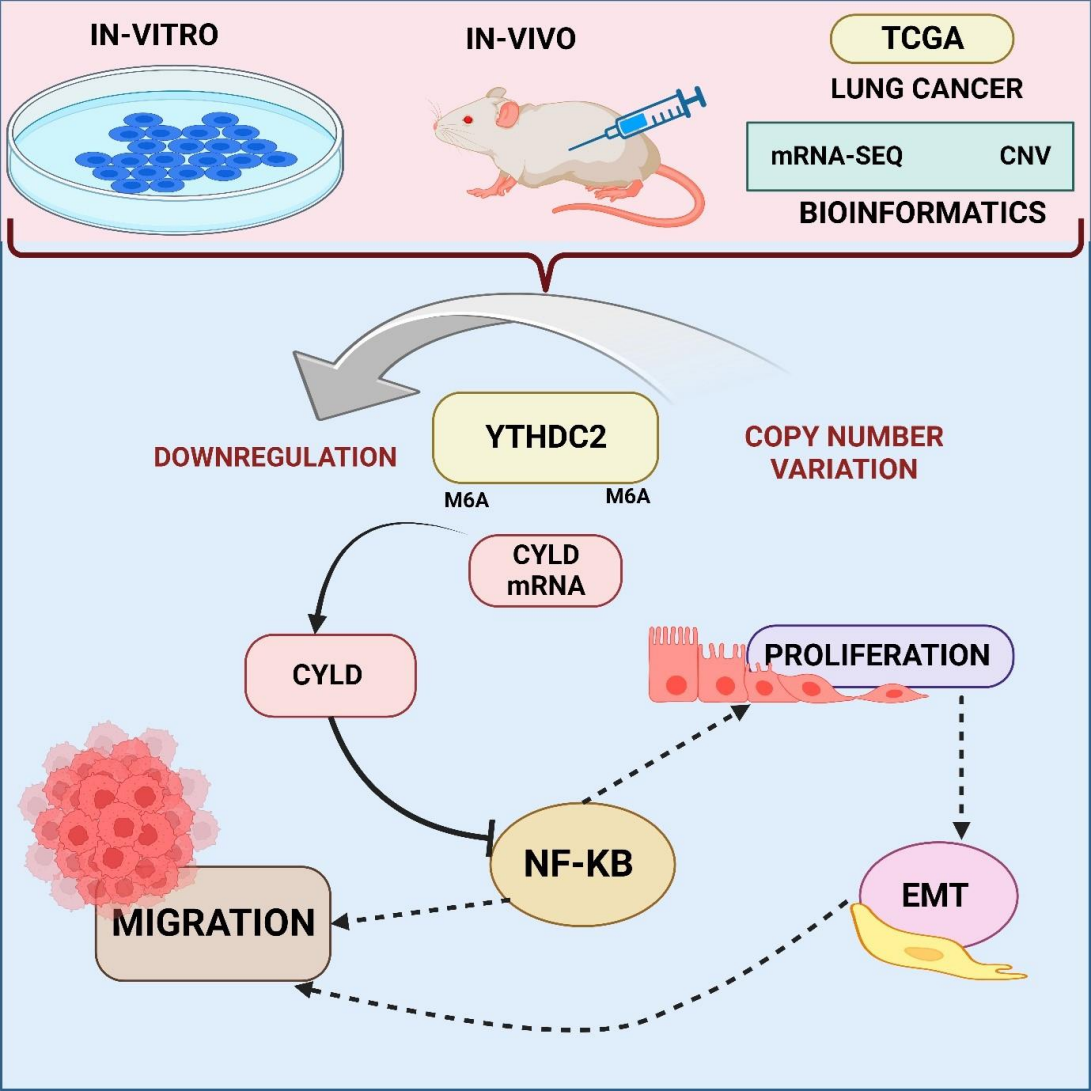
This image reveals that lung cancer-exposed cells express less YTHDC2, linked to smoking history and poor prognosis. YTHDC2 overexpression inhibits cancer *in vivo*, influenced by copy number deletion, and m6A alteration mediates its tumor-suppressive role via the CYLD/NF-κB pathway. A vital connection is established between smoking-induced YTHDC2 downregulation and accelerated lung cancer development.

Lung cancer caused by smoking is largely influenced by NF-κB. The transcription factor NF-κB, which controls the expression of genes linked to cell division, survival, and inflammation, is activated by prolonged inhalation of tobacco smoke (Rojas-Quintero et al., 2023[166]). A number of processes are triggered by this activation, which contributes to the tumor's development and dissemination (Sivandzade et al., 2019[174]). NF-κB promotes angiogenesis, prevents apoptosis, and helps cancer cells survive (Sokolova and Naumann, 2017[175]). Moreover, it sets off the production of cytokines that promote inflammation, creating an environment that is conducive to the growth of tumors (Thapa et al., 2022[187]; Walter et al., 2020[190]). Targeting NF-κB signaling pathways presents a promising avenue for therapeutic therapies in smoking-associated lung cancer by interfering with critical processes that promote oncogenesis and progression (Bhat et al., 2024[22]; Wang et al., 2019[191]). Cellular signaling and cytoskeletal regulation are mediated by a protein known as MARCKS (Wei et al., 2022[195]). It is necessary for a number of biological processes, including as cell motility, membrane trafficking, and exocytosis (Wronka et al., 2022[198]). Numerous physiological and pathological processes in cells are impacted by MARCKS's interaction with cell membranes, which is influenced by its level of phosphorylation (Li et al., 2019[119]). Liu et al. examined the function of MARCKS in lung cancer caused by smoking. It was discovered that smoking cigarettes activates MARCKS in lung cancer cells and the epithelium of the airways. The NF-κB signaling is activated as a result of this activation, and low survival and phospho-NF-κB are strongly associated. On the other hand, EMT, pro-inflammatory cytokines, and stem-like characteristics are all upregulated by phospho-MARCKS. These effects are suppressed when MPS peptide is used to target MARCKS phosphorylation (Liu et al., 2021[123]) (Figure 3(Fig. 3)).

### Cell surface receptor-mediated pathways

The cell surface receptor for IGF-1 is IGF-1R. It relates to the family of receptor tyrosine kinases and is crucial for cellular growth, differentiation, and survival (Bhalla et al., 2022[17]; Geffken et al., 2022[66]). IGF-1R activation sets off a signaling cascade that controls a volume of cellular functions and aids in the growth and upkeep of tissues (Hellstrom et al., 2017[77]). IGF-1R signaling dysregulation has been linked to cancer and other illnesses (Higashi et al., 2019[78]). According to the study by Min et al., smoking-related lung carcinogenesis is connected with the activation of RTKs. The study revealed that this process requires NNK-induced activation of the β-AR, which in turn promotes the IGF-1R. NNK administration activated the IGF-1R signaling pathway, which was blocked by pharmacologic or genetic suppression of β-AR. β-AR and NF-κB agonists were shown to increase IGF-1R phosphorylation, and treating mice with β-AR antagonists prevented the development of lung cancers. In order to help smokers, avoid lung cancer, the research suggests that blocking β-AR-mediated IGF-1R activation might be an effective strategy (Min et al., 2016[139]). Tpl2 kinase is a serine-threonine kinase that is involved in inflammatory and immunological responses (Escós et al., 2022[59]). It is sometimes referred to as Cot kinase. It has a role in MAP kinase pathway activation (Bhat et al., 2023[23]; Johannessen et al., 2010[96]). A study by Sun et al. discovered that NF-κB actually has a tumor-suppressive effect. Low patient survival and elevated exposure to cancer of the lungs in people are associated with NF-κB1 downregulation. NF-κB1-deficient mice, however, are more susceptible to lung carcinogenesis brought on by urethane, a smoking carcinogen. Instead of acting as an NF-κB factor, as is the case with its traditional role, NF-κB1 suppresses tumors via stabilizing Tpl2 kinase. When it comes to lung carcinogenesis triggered by urethane, Tpl2-knockout animals are similar to NF-κB1 knockouts. The study revealed unidentified role for p105/ Tpl2 signaling in lung homeostasis and an unexpected, NF-κB-independent but Tpl2-dependent role for NF-κB1 in suppressing lung tumors (Sun et al., 2016[177]). 

One cell surface receptor connected to inflammatory reactions is the RAGE (Krizan and Johar, 2015[108]). Cigarette smoke exposure raises the expression of RAGE, which exacerbates oral inflammation (Leone et al., 2021[115]). It was investigated if semi-synthetic glycosaminoglycan ethers (SAGEs) could suppress RAGE signaling. In gingival epithelial cells, co-treatment with SAGEs and cigarette smoke extract reduced inflammatory responses (MacLaren, 2015[130]). This implies that SAGEs could be useful in reducing the effects of RAGE-mediated inflammation, offering a possible path for therapeutic treatments in illnesses made worse by exposure to tobacco smoke (Rasmussen, 2015[159]). Sanders et al. examined that exposing gingival Ca9-22 to 10 % CSE for six hours increased RAGE expression and activated NF-κB and Ras signaling. Elevated IL-6 and IL-1β in the culture medium indicated inflammatory responses. SAGEs were used to block RAGE signaling, reducing inflammatory effects when co-treated with CSE. This suggests a potential therapeutic target in NF-κB-related pathways for diseases exacerbated by tobacco smoke-induced oral inflammation (Sanders et al., 2017[168]).

### Genetic variants and polymorphisms

Genetic variants in the NF-κB1 gene, which codes for a subunit of the NF-κB transcription factor, are referred to as NF-κB1 polymorphisms (Aidinidou et al., 2021[3]). These differences may impact NF-κB activity, which in turn may impact inflammation and the immune system (Bhat et al., 2023[23]; Cartwright et al., 2016[30]). Among other diseases, certain SNPs have been connected to a higher chance of developing autoimmune and inflammatory disorders (Jin et al., 2019[95]). Yin et al. studied five different forms of NF-κB1, which were investigated in relation to smoking and other genes in research that included 387 Chinese controls that were cancer-free, whereas 384 patients had lung cancer. Although individual variations did not directly correlate with the risk of lung cancer, a particular haplotype did indicate a lower risk when smoking duration was taken into account. The results emphasize the importance of NF-κB and smoking in inflammation-associated lung cancer etiology and point to a possible link between NF-κB1 polymorphisms, smoking duration, and lung cancer development in the Chinese population (Yin et al., 2015[203]). 

The study by Lu et al. on the effects of CSE on HBE cells also showed that CSE decreased PTEN levels while increasing the expression of miR-21, p-Akt, p-NF-κB and HIF-1α. Suppression of PTEN caused by increased miR-21 has an effect on the Akt/NF-κB pathway. Silencing of HIF-1α or NF-κB prevented CSE-induced alterations, whereas miR-21 up-regulation reversed these effects. The results uncover a new pathway that connects smoking, miR-21, HIF-1α, and NF-κB to lung carcinogenesis brought on by CSE (Lu et al., 2018[126]). Using a squamous cancer cell line as a comparison, Rohrer et al. examined the effect of CSC on NF-κB activation in HPV-transformed oral and bronchial cells. NF-κB was activated in a dose-dependent manner by all cells, while HPV-transformed cells responded more strongly. Smoking cigarettes may accelerate the NF-κB-dependent development of HPV+ premalignancy into cancer. The findings propose that future research should concentrate on NF-κB targeting during premalignancy and malignant development, especially in HPV+ individuals (Rohrer et al., 2010[165]).

While the NF-κB1 rs28362491 polymorphism is linked to altered NF-κB activity, a crucial regulator of inflammation and immunological responses, the PPP1R13L gene produces a protein implicated in apoptosis (Chen et al., 2016[34]; Guo et al., 2021[71]). Genetic differences in NF-κB1 may impact NF-κB-mediated activities, which in turn may affect susceptibility to certain illnesses (Li and Zhang, 2019[118]). Comprehending these genetic variables offers valuable perspectives on the intricate relationship between genetic alterations and cellular processes in both well-being and illness (Long et al., 2022[125]). Yin et al. evaluate the relationship between smoking duration, PPP1R13L, NF-κB1 rs28362491 polymorphism, and lung cancer risk in a Chinese population. After examining 544 instances of lung cancer and 550 age, sex, and ethnicity-matched controls, NF-κB1 rs28362491 was not associated with the risk of developing lung cancer. A higher risk was seen in the dominant model for CD3EAP rs967591. Lung cancer was linked to a common haplotype comprising CD3EAP rs967591(A), PPP1R13L rs1970764(G), and CD3EAP rs735482(C). The results of interaction studies demonstrated associations between the length of smoking and NF-κB1 rs28362491-PPP1R13L rs1970764 as well as CD3EAP rs735482, indicating participation in the same pathway that results in smoking-induced lung cancer (Yin et al., 2016[202]). 

### Interaction with nicotine and NNK

Cigarette Smoke Extract (CSE) is produced when cigarette smoke condenses, it is made up of a complicated mixture of substances, such as tar, nicotine, and several hazardous compounds (Cai et al., 2021[29]). In order to imitate the effects of cigarette smoke exposure on biological systems, researchers frequently utilize CSE in their studies (Fang et al., 2021[61]). This helps to identify possible therapies for smoke-induced health concerns as well as insights into the processes behind smoking-related disorders (Gellner et al., 2016[67]). Zhao et al. examined the effects of CSE on HBE cells, with particular attention to the transition between the epithelium and mesenchymal tissue (EMT) and inflammation. Exposure to CSE for an extended period of time resulted in EMT and cellular transformation, which in turn activated NF-κB and increased proinflammatory interleukin-6 (IL-6). As a result, miR-200c levels dropped. Suppressing miR-200c and boosting EMT, NF-κB and IL-6 pathways were shown to be involved in CSE-induced IL-6 elevation. The maintenance of the change produced by CSE required IL-6. The prevention of CSE-induced EMT and malignant transformation was achieved by NF-κB inhibition, revealing a connection between inflammation, EMT, and CSE-induced lung carcinogenesis (Hussain et al., 2023[92]; Zhao et al., 2013[209]). 

Nicotine is a stimulant that affects the central nervous system and is extremely addictive (Bitzer et al., 2023[25]; Brown et al., 2003[28]). Tobacco smoke contains NNK, a powerful carcinogen (Ding et al., 2008[52]). The body activates NNK metabolically, which damages DNA and promotes the growth of certain malignancies, including lung cancer (Edwards et al., 2021[56]). The negative health consequences of tobacco usage are mostly caused by both nicotine and NNK (Gankhuyag et al., 2017[64]; Singla et al., 2023[173]). Tsurutani et al. examined the effects of nicotine and NNK, two tobacco constituents, on the survival and proliferation of lung cancer cells via triggering the Akt pathway. In both SCLC and NSCLC cells, nicotine and NNK quickly activated Akt. FKHR, GSK-3, mTOR, tuberin, and S6K1 were among the downstream substrates whose phosphorylation was elevated by nicotine. Different nicotinic acetylcholine receptor subunits were expressed by NSCLC cells. Akt was activated by nicotine via the alpha3/alpha4 or alpha7 subunits. In an Akt-dependent and NF-κB-dependent way, nicotine-induced proliferation and prevented apoptosis, pointing to a possible connection between smoking and the advancement of cancer (Tsurutani et al., 2005[188]). Similarly, Sugano et al. looked at how nicotine affected the inflammatory mediators that activated macrophages generated. Before LPS treatment, nicotine dramatically reduced the transcriptional levels of PGE2, IL-1, and IL-8 in U937 cells. The decrease of NF-κB, a transcription factor essential for activating these genes, was connected to the inhibition. These results imply that nicotine's inhibition of NF-κB may be a factor in the immunosuppression brought on by cigarette smoke (Sugano et al., 1998[176]).

A type of cigarette called light cigarettes (LC) is made to emit less tar and nicotine when smoked (Benowitz et al., 2017[15]). For this, manufacturers employ filters, modified tobacco mixes, or a combination of the two (Breitbarth et al., 2018[27]). Even though they are referred to be "light," smokers may make up for lower nicotine levels by taking larger puffs or smoking more cigarettes, so they are not always a safer option (Chang et al., 2021[31]; Thapa et al., 2024[180]). There are still health concerns, and regulations have been put in place to clear up misunderstandings about how little harm they actually cause (Crosbie et al., 2022[40]). Valenca et al. show that exposure to light cigarettes (LC) is thought to carry less health hazards than conventional cigarettes; nonetheless, research conducted on C57BL/6 mice showed negative consequences after 60 days of exposure to 3, 6, or 12 LC. Emphysema, an imbalance in ECM, a reduction in tissue inhibitor of metalloprotease-2, and an increase in matrix metalloprotease-12 detection were seen in the 12 LC group. Despite thoughts of less danger, the study indicates that LC may really be harmful to the lungs, altering the balance of the extracellular matrix and exacerbating emphysema. In comparison to mice exposed to ambient air, higher levels of NF-κB and TNF-alpha were also seen in the LC-exposed groups (Valenca et al., 2006[189]).

### Role of specific pollutants in NF-κB activation

Sulfur-containing fossil fuels are the main source of sulphur dioxide (SO_2_), a colorless gas with a strong odor. It has a role in acid rain generation and air pollution (Blum et al., 2023[26]; Chen et al., 2022[36]). Inhaling SO_2_ can cause respiratory problems, including difficulty breathing. It is a major air pollutant that is controlled to safeguard the public's health and the environment (Guo et al., 2021[70]). Yun et al. showed at low doses, their synergistic toxicity was shown, leading to considerable impairment in cell survival and apoptosis. Similar to how NF-κB plays a role in smoking-induced lung cancer, the mechanism included the generation of ROS and the activation of NF-κB, highlighting the need of taking into account interactions between pollutants (Yun et al., 2015[204]). 

### COX pathways and proinflammatory mediators

The enzyme cyclooxygenase-2 (COX-2) is in charge of producing prostaglandins, which are inflammatory mediators (Bernard et al., 2008[16]). In contrast to COX-1, COX-2 is induced and frequently increased in response to stimuli that cause inflammation (Doré, 2011[53]). Because COX-2 overexpression is linked to a number of illnesses, such as cancer and inflammatory ailments, it is a target for therapeutic therapies (Frejborg et al., 2020[63]). Ratovitski et al. examined the effect of cigarette smoke on the transcriptional control of inflammatory molecules, with NF-κB being the main emphasis. When primary and secondary smoking were modeled using extracts from mainstream and sidestream smoke, it was shown that there was dose-dependent downregulation of LKB1 and overexpression of PEA3 and COX-2 proteins. Endogenous ChIP study demonstrated that NF-κB binds to the COX-2 promoter and forms compounds in lung tumor cells exposed to cigarette extracts. These findings underscore the significance of inflammation in the carcinogenesis of epithelial cells by highlighting a unique relationship between transcription factors, most notably NF-κB, and the cellular response to cigarette smoke (Ratovitski, 2010[161]). Further, Baglole et al. show that compared to wild-type (AhR(+/+)) fibroblasts, lung fibroblasts without the AhR gene (AhR(-/-)) show an increased inflammatory reaction to smoking from cigarettes, which is characterized by higher levels of prostaglandins (PGs) and COX-2. Transient transfection was used to validate AhR expression, and it showed a substantial reduction in smoke-induced COX-2 and PG production in AhR(-/-) fibroblasts, indicating the anti-inflammatory activity of AhR. Although AhR and NF-κB interact, the increased inflammatory response in AhR(-/-) fibroblasts is linked to RelB loss rather than NF-κB (p50/p65) activation. This emphasizes AhR and RelB's possible therapeutic target in treating disorders associated with inflammation (Baglole et al., 2008[9]). 

Specialized immune cells called sentinel cells serve as the body's first line of defense against infections or other dangers (Daigneault et al., 2010[42]; Gupta et al., 2023[72]). Because these cells are always keeping an eye on their environment, they are essential to the immune system's surveillance (Hodge et al., 2022[83]). Sentinel cells sense foreign invaders or aberrant cells, and when they do, they trigger an immunological response that activates other immune system components to get rid of the threat and keep the body healthy overall (Marciscano and Anandasabapathy, 2021[133]; Pitt et al., 2016[151]). Martey et al. investigated the significance of fibroblasts, specifically their function as "sentinel" cells that initiate inflammation by releasing proinflammatory chemicals such as prostaglandins. Through the promotion of chronic inflammation, the overexpression of COX-2 and excessive eicosanoid synthesis, which are found in early carcinogenesis, may establish a susceptibility to cancer. When normal human lung fibroblasts are exposed to cigarette smoke, NF-κB activation-mediated prostaglandin production and COX-2 expression are elevated. This may be the first stage of epithelial transition, this persistent inflammatory condition (Martey et al., 2004[134]).

### Role of Rel subtypes and alternative NF-κB pathways

RelB is a transcription factor that belongs to the NF-κB family and is involved in immune and inflammatory responses (Kim et al., 2023[103]; Millet et al., 2013[138]). RelB is not like other NF-κB proteins in that it frequently suppresses the immune system (Mockenhaupt et al., 2021[140]). It controls genes involved in immunological tolerance and the development of lymphoid organs and creates alternative NF-κB dimers (Navarro et al., 2023[144]). RelB is a possible therapeutic target for regulating immune responses since it is linked to a number of illnesses (Seet et al., 2021[169]). The function of RelB in controlling smoke-induced COX-2 protein production in pulmonary fibroblasts is examined by Zago et al. RelB deficiency reduces the production of COX-2 protein even in the presence of Cox-2 mRNA increase and activation of the NF-κB pathway. The results show that whereas RelB has no effect on mRNA stability, it does have an effect on miR-146a baseline levels. Smoking cigarettes raises miR-146a only in cells that express RelB. While inhibiting miR-146a greatly increases COX-2 protein levels, it has little effect on RelB or Cox-2 mRNA stimulation by smoke. Accordingly, it is possible that a novel pathway, the RelB-miR-146a axis, regulates inflammation in reaction to pulmonary toxicants, particularly those associated with NF-κB and smoking (Zago et al., 2014[205]).

In lung tissues, the carcinogens in CS, such as nitrosamines and polycyclic aromatic hydrocarbons, cause inflammatory reactions and genetic alterations (Armstrong et al., 2004[5]; Holme et al., 2023[85]; Thapa et al., 2023[186]). Prolonged exposure triggers the activation of oncogenic pathways, such as NF-κB, which facilitates angiogenesis, cell survival, and proliferation (Kordiak et al., 2022[105]). Furthermore, the inflammation brought on by CS fosters the growth of tumors (Moorthy et al., 2015[141]). These interrelated factors support the development and spread of lung cancer, highlighting the crucial part that cigarette smoke plays in the etiology of this fatal illness (Petit et al., 2019[150]; Singh et al., 2018[172]). Yao et al. investigate the mechanism by which lung cancer in mice is produced by CS. RelA/p65⁻/⁻ mice and WT mice were split into two groups and given either air or CS. When compared to WT mice exposed to air, CS-treated animals displayed increased lung weight, tumor multiplicity, and maximal tumor size. WT mice's longevity was markedly shortened by CS exposure, but myeloid cells with RelA/p65 deleted had a higher survival rate. Increased macrophage infiltration and higher Ki-67-positive cells were linked to CS-induced lung cancer development. According to the study, TNFα and the myeloid cell RelA/p65 are important factors in the development of lung cancer brought on by CS (Yao et al., 2014[201]) (Table 1(Tab. 1); References in Table 1: Baglole et al., 2008[9]; Jin et al., 2019[95]; Liu et al., 2021[123]; Lu et al., 2018[126]; Ma et al., 2015[128]; Majumder et al., 2021[131]; Min et al., 2016[139]; Ratovitski, 2010[161]; Rioux and Castonguay, 2000[163]; Rohrer et al., 2010[165]; Sugano et al., 1998[176]; Sun et al., 2016[177]; Valenca et al., 2006[189]; Wang et al., 2021[192]; Yin et al., 2016[202]; Yun et al., 2015[204]; Zago et al., 2014[205]; Zhang et al., 2018[208]).

## Epigenetic Mechanisms in Smoking-Induced NF-κB Activation

### METTL3 and m6A modification

METTL3 functions as a methyltransferase in the critical RNA regulatory process of N6-methyladenosine (m6A) modification (Chen et al., 2022[32]). On mRNA, it adds m6A markers. The m6A reader protein YTHDF2 mediates the degradation of mRNA (Chen et al., 2022[33]; Li et al., 2022[120]). YTHDF2 controls the activity of METTL3, which has an impact on translation and mRNA stability (Sun et al., 2023[179]). The dynamic interaction between YTHDF2 and METTL3 plays a major role in controlling post-transcriptional gene expression in cells (Wang et al., 2022[194]). Jin et al. showed smoking cigarettes causes aberrant m6A alteration, which is connected to the development of NSCLC. The m6A METTL3 and the m6A YTHDF2 facilitate this change, which results in a reduction in the stability and expression of DAPK2 mRNA. The m6A METTL3 and m6A YTHDF2 mediate this change. Additionally, the study shows that DAPK2 suppresses tumors and that its downregulation increases the capacity for migration and proliferation by triggering the NF-κB signaling pathway. The results show that smoking cigarettes alters DAPK2, which may be a therapeutic target for patients with NSCLC caused by smoking and provide new information on the mechanisms behind the development of NSCLC (Jin et al., 2021[94]). Similarly, According to Wang et al.'s analysis, cells exposed to lung cancer and cigarettes expressed less of the m6A reader protein YTHDC2. The relationship between lower YTHDC2, smoking history, and a bad prognosis is demonstrated via tissue analysis and bioinformatics. *In vivo*, confirmation of YTHDC2 overexpression inhibits lung cancer cell migration and proliferation. In lung cancer, YTHDC2 decrease is modulated by copy number deletion. Notably, m6A alteration mediates YTHDC2's action as a tumor suppressor via the CYLD/NF-κB pathway, indicating a crucial connection between smoking-related YTHDC2 downregulation and accelerated lung cancer development (Wang et al., 2021[192]) (Figure 3(Fig. 3)).

### Histone modifications

Histone modifications, such as acetylation, methylation, and phosphorylation, play a crucial role in regulating chromatin structure and gene expression. NF-κB activation in response to smoking can lead to changes in histone acetylation and methylation patterns, which affect the transcriptional activity of various genes involved in inflammation and tumorigenesis (Adcock et al., 2006[2]; Barnes, 2011[14]; Kaur et al., 2022[100]). Smoking-induced NF-κB activation can increase histone acetylation at specific gene promoters, enhancing the expression of pro-inflammatory cytokines and other oncogenic factors (Kaur et al., 2018[100]). Histone acetyltransferases (HATs) like p300/CBP are recruited to the promoters of NF-κB target genes, leading to increased acetylation of histones H3 and H4, which promotes gene transcription. Conversely, histone deacetylases (HDACs) can be involved in repressing the transcription of tumor suppressor genes. Smoking can disrupt the balance between HATs and HDACs, leading to aberrant gene expression patterns that favor cancer progression (Cui et al., 2013[41]).

### DNA methylation

DNA methylation is another epigenetic mechanism influenced by NF-κB activation in smoking-induced lung cancer. NF-κB can interact with DNA methyltransferases (DNMTs) to modulate the methylation status of specific gene promoters (Asgarova et al., 2018[7]; Barnes, 2011[14]). Hypermethylation of tumor suppressor genes and hypomethylation of oncogenes are common features observed in smoking-related lung cancer (Kaur and Batra, 2020[99]). The promoter regions of tumor suppressor genes, such as p16 and RASSF1A, can become hypermethylated in response to chronic smoking, leading to their silencing and subsequent loss of tumor-suppressive functions (Liu et al., 2023[124]). NF-κB-mediated recruitment of DNMTs to these promoters contributes to the epigenetic silencing of crucial regulatory genes, promoting cancer development (Xiong et al., 2019[200]).

### Non-coding RNAs

**Figure 4:**
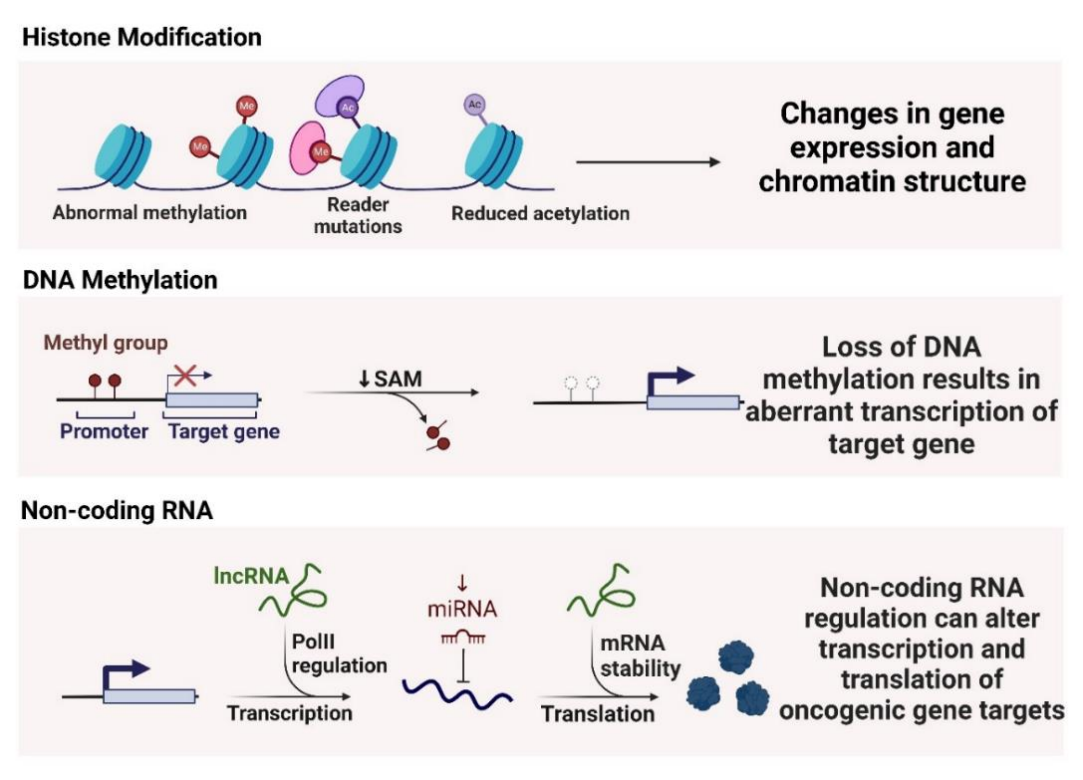
The figure illustrates epigenetic mechanisms in cancer: Histone modifications lead to changes in gene expression and chromatin structure; DNA methylation loss results in aberrant transcription of target genes; Non-coding RNA regulation alters transcription and translation of oncogenic gene targets.

Non-coding RNAs, including microRNAs (miRNAs) and long non-coding RNAs (lncRNAs), are also regulated by NF-κB and play significant roles in smoking-induced lung carcinogenesis (Barangi et al., 2023[13]). NF-κB can modulate the expression of various miRNAs that act as either oncogenes or tumor suppressors. miR-21, an oncogenic miRNA, is upregulated in response to smoking-induced NF-κB activation (Chen et al., 2021[35]). MiR-21 targets and downregulates tumor suppressor genes such as PTEN, leading to enhanced cell survival and proliferation (Elton et al., 2013[57]). Conversely, tumor-suppressive miRNAs like miR-34a can be downregulated by smoking, further contributing to lung cancer progression (Kolenda et al., 2020[104]). LncRNAs, such as MALAT1, are also involved in NF-κB-mediated epigenetic regulation. MALAT1 has been shown to interact with NF-κB and other chromatin-modifying proteins to influence gene expression patterns associated with inflammation and cancer (Goodarzi et al., 2023[69]) (Figure 4(Fig. 4)).

## Therapeutic Interventions

Extensive research has focused on elucidating NF-κB's role in smoking-induced lung cancer. Activated by cigarette smoke, NF-κB emerges as a pivotal molecular player, fostering tumorigenesis and progression (Hasnis et al., 2007[75]). Cigarette smoke triggers sustained NF-κB nuclear translocation, prompting the release of inflammatory mediators and heightened mucus cell secretion (Huang et al., 2022[89]). This smoking-induced NF-κB activation intricately regulates downstream pathways linked to lung cancer (Mei et al., 2022[137]; Thapa et al., 2024[180]). Additionally, ongoing investigations explore NF-κB inhibition as a potential therapeutic avenue for smoking-associated lung cancer (Ou et al., 2022[145]). A comprehensive understanding of the NF-κB pathway's involvement in tobacco smoke-induced lung cancer holds promise for informing the development of effective therapeutic interventions (Preciado et al., 2008[152]).

### Natural compounds

Linalool is a naturally occurring terpene alcohol found in many flowers and spice plants (An et al., 2021[4]). It is classified as an acyclic monoterpenoid and has multiple commercial applications, primarily based on its pleasant scent, which is described as floral with a touch of spiciness (de Groot, 2019[44]). Linalool is a colorless oil and is used in the manufacturing of soaps, fragrances, and other products (Dos Santos É et al., 2022[54]). It is also hydrogenated to give dihydro- and tetrahydrolinalool, which are more resilient fragrances (Jiang et al., 2023[93]). Linalool has various biological properties, including antimicrobial activity (Koriem and El-Qady, 2023[106]). In recent years, there has been interest in using linalool as an active packaging, for example, by injecting it into the space above the contents of food packaging or applying it to food (Levenberg et al., 2020[117]). Linalool was found to significantly reduce lung inflammation caused by chronic sclerosis in mice by Ma et al. (2015[128]). It prevented inflammatory cell infiltration and the generation of inflammatory cytokines like IL-6, TNF-α, IL-8, IL-1β, and MCP-1. Linalool also prevented lung MPO activity and pathological alterations. Furthermore, it dose-dependently inhibited the activation of NF-κB produced by chronic sclerosis, thereby reducing chronic sclerosis-induced lung inflammation (Ma et al., 2015[128]; Thapa et al., 2023[182]). 

Bromelain is an enzyme extract derived from the stems of pineapples, although it exists in all parts of the fresh pineapple (Arshad et al., 2014[6]). It is a complex combination of multiple endopeptidases of thiol and other compounds (Chobotova et al., 2010[38]). Bromelain has a history of folk medicine use and is currently used in various industries, such as cosmetics, as a topical medication, and as a meat tenderizer (Hikisz and Bernasinska-Slomczewska, 2021[79]; Thapa et al., 2023[183]). It has been studied for its various therapeutic applications, including its role as an antiedematous, fibrinolytic, anticancer, anti-inflammatory, antibiotic, anticoagulant, and antithrombotic agent (Hirche et al., 2020[81]; Kumar et al., 2023[109]). Bromelain has been shown to have anticancer effects by preventing nuclear factor κB (NF-κB) translocation and inducing G2/M arrest, which can disrupt normal apoptotic functions and help cancer cell transformation (Maurer, 2001[135]; Owoyele et al., 2020[146]). The study of Majumder et al. explored the impact of bromelain and ethanolic olive leaf extract on lung carcinogenesis. They found that the combination increases Nrf2 activity, decreases NF-κB translocation, and regulates inflammatory indicators, making it a potential method to reduce lung cancer caused by smoking (Majumder et al., 2021[131]). 

Diallyl trisulfide (DATS) is an organosulfur compound found in garlic, known for its various biological activities, particularly in the context of cancer prevention and treatment (Han et al., 2017[74]). It has been reported to selectively kill cancerous cells in the prostate and breast while leaving healthy cells unharmed, attributed to increased reactive oxygen species (ROS) within cancer cells (Hsieh et al., 2020[88]; Kanga et al., 2023[98]). DATS has been investigated as an anticancer and chemopreventive agent, with *in vitro* studies showing its potency in reducing cell viability and its lower toxicity to non-transformed cells (Kim et al., 2023[102]; Leung et al., 2020[116]). Additionally, DATS exhibits a range of activities, including anticancer chemotherapeutic (Lin et al., 2024[121]), immunostimulatory (Puccinelli and Stan, 2017[154]), antioxidative (Rauf et al., 2022[162]), hepatoprotective, anti-fibrotic, anti-estrogenic (Wu et al., 2011[199]), anti-metastatic, anti-inflammatory, and anti-angiogenic properties (Thapa et al., 2023[186]; Zeng et al., 2008[206]). It has also been shown to modulate disease states such as cancer, infection, and metabolic syndrome. Qu et al. found that garlic oil's DATS can reduce lung carcinogenesis induced by tobacco-specific nitrosamine NNK. When injected into an A/J mouse model, DATS significantly reduced lung tumor incidence. DATS enhanced *F. rodentium* bacteria in the gut microbiome, inhibiting tumor growth. It also inhibited inflammatory factors and negatively regulated NF-κB signaling through the PPARγ pathway, suggesting DATS as a potential new chemopreventive treatment for lung cancer caused by tobacco use (Qu et al., 2024[157]).

### Pharmaceutical drugs

Celecoxib has been evaluated as a potential cancer chemopreventive and therapeutic drug in clinical trials for various types of cancer (Bąk and Krupa, 2023[10]; Thapa et al., 2023[184]). It has been shown to have anticancer properties by preventing nuclear factor κB (NF-κB) translocation and inducing G2/M arrest, which can disrupt normal apoptotic functions and help cancer cell transformation (Fidahic et al., 2017[62]). Celecoxib is extensively metabolized through cytochrome P450 2C9 (CYP2C9) and may have interactions with other medications (Krasselt and Baerwald, 2019[107]; Papageorgiou et al., 2016[149]). Shishodia et al. studied NF-κB activation in lung cancer cells exposed to CSC. They found that celecoxib reduced persistent NF-κB activation for up to 24 hours, inhibiting the activation, phosphorylation, degradation, and nuclear translocation of I-κB elicited by CSC. Additionally, it inhibited the expression of genes reliant on NF-κB, reducing inflammation, proliferation, and angiogenesis (Shishodia and Aggarwal, 2004[170]).

Angiotensin-(1-7) (Ang-(1-7)) is a vasoactive peptide of the renin-angiotensin system (RAS), which is generated mainly by angiotensin-converting enzyme 2 (ACE2) and exerts its actions via the Ang-(1-7)/ACE2/Mas axis (de Paula Gonzaga et al., 2020[45]). It is considered to be a main mechanism that counterbalances the vasoconstrictive actions of the classical RAS. Ang-(1-7) has been reported to release nitric oxide, induce diuresis, and have a vasodilatory effect, favoring a lowering of blood pressure (de Souza-Neto et al., 2018[47]; Lelis et al., 2019[114]). It is a heptapeptide found in the heart and kidneys, with concentrations six times higher in the kidney than in plasma, highlighting its role in renal homeostasis (Luna et al., 2023[127]). Ang-(1-7) has broad effects in the cardiovascular system, including vasodilatation, myocardial protection, antiarrhythmic, antihypertensive, and positive inotropic effects, as well as inhibition of pathological cardiac remodelling (Medina and Arnold, 2019[136]; Pan et al., 2018[147]). It also has favourable effects on metabolism by lessening insulin resistance. Ang-(1-7) is considered to be an endogenous ligand for the G protein-coupled receptor Mas (Rahimi et al., 2022[158]). Zhang et al. studied Angiotensin-(1-7) in relation to cigarette smoke-induced COPD in C57BL/6J mice. Although Ang-(1-7) could not stop emphysema and lung function deterioration caused by cigarette smoke, it successfully reduced lung fibrosis and inflammation. The study suggests Ang-(1-7) has potential in treating emphysema and lung function impairment, possibly through the NF-κB pathway (Zhang et al., 2018[208]). 

NSAIDs lower fever, lessen inflammation, and relieve pain. They function by preventing prostaglandins, which are molecules involved in the inflammatory response, from being produced (Bindu et al., 2020[24]; Ding, 2022[51]). NSAIDs, which are often used to treat ailments including arthritis, include aspirin, ibuprofen, and naproxen (García-Rayado et al., 2018[65]). NSAIDs are useful, but they should be used carefully since they can cause gastrointestinal discomfort and should be taken for extended periods or at high doses (Gurpinar et al., 2014[73]; Panchal and Prince Sabina, 2023[148]). Rioux et al. found that NSAIDs can prevent lung tumor growth when NNK, a smoking-related carcinogen, is present. They found that COX-1 and -enzymes mediate the conversion of NNK into carcinogenic substances in human macrophages. NSAIDs, like acetylsalicylic acid and NS-398, decreased COX-dependent NNK activation by 66 % and 37 %, respectively. This suggested that inhibiting NF-kappaB activation may be a unique way to prevent lung cancer (Rioux and Castonguay, 2000[163]; Thapa et al., 2023[185]). 

## Challenges and Future Directions

Lung cancer growth is specifically impacted by the precise processes of NF-κB activation in response to stress caused by smoking, which are yet not fully understood. While it is known that cigarette smoke can activate NF-κB, the detailed interplay between NF-κB and other signaling pathways implicated in lung cancer development remains a subject of ongoing research. Not all smokers develop lung cancer, and the disease can also arise in lifetime non-smokers, indicating a complex interplay of genetic and environmental factors. Understanding the variability in disease susceptibility and the specific role of NF-κB in this context is a significant challenge. The variation in disease susceptibility has stimulated interest in molecular epidemiologic investigations to better understand the molecular mechanisms underlying the link between smoking, NF-κB, and lung cancer development.

Further research must focus on elucidating the exact mechanisms by which NF-κB is activated in response to smoking-related stress, as well as its distinct roles in the development and progression of smoking-related lung cancer. Personalized therapy solutions will require the completion of extensive molecular epidemiology research to determine genetic and environmental variables that modify NF-κB activity and its connection with smoking-induced lung cancer. It has potential to develop tailored therapy techniques that modify NF-κB activity in lung cancer caused by smoking. This involves investigating drugs that can precisely target the NF-κB signaling system, such as NF-κB inhibitors, for the treatment and prevention of lung cancer caused by smoking.

## Conclusion

In overview, study of the function of NF-κB in smoking-induced lung cancer has shown a complicated web of molecular processes that play a role in the onset and spread of this deadly illness. This review has compiled important data and insights from a thorough investigation of the literature, illuminating the complex relationship between NF-κB and the toxic breath of smoking-induced lung cancer. NF-κB, a transcription factor with diverse cellular functions, emerges as a central orchestrator in the response to cigarette smoke exposure. Its activation is intricately linked to chronic inflammation, immune modulation, and cell survival - all pivotal processes in the pathogenesis of lung cancer. A thorough examination of NF-κB's unique contributions is crucial because of the milieu that is favorable to cancer created by the interaction between this protein and the several carcinogens found in cigarette smoke. The pro-inflammatory and pro-survival activities of NF-κB in the context of smoking-induced lung cancer underline its potential as a therapeutic target. Via clarifying the molecular pathways via which NF-κB affects the development of cancer, scientists are able to identify important targets for intervention and prevention.

## Declaration

### Ethics approval and consent to participate

Not applicable to a review article. 

### Consent for publication 

All authors gave their consent for publication. 

### Availability of data and materials 

Not applicable as no novel data were generated for this review article. 

### Competing interests 

No authors have any conflict of interest or competing interests to declare. 

### Funding 

No funding was received to perform this study. 

### Author contributions 

RT, EM, and AG researched the data. AAB, WHA, IK, SIA, and HA wrote the first draft of the manuscript. BGO, RM, HD, SKS, and GG edited the manuscript. KD was responsible for conceptualization and supervision. All authors reviewed and approved the final version of the manuscript. Kamal Dua is the guarantor of this work.

### Acknowledgments 

None.

## Figures and Tables

**Table 1:**
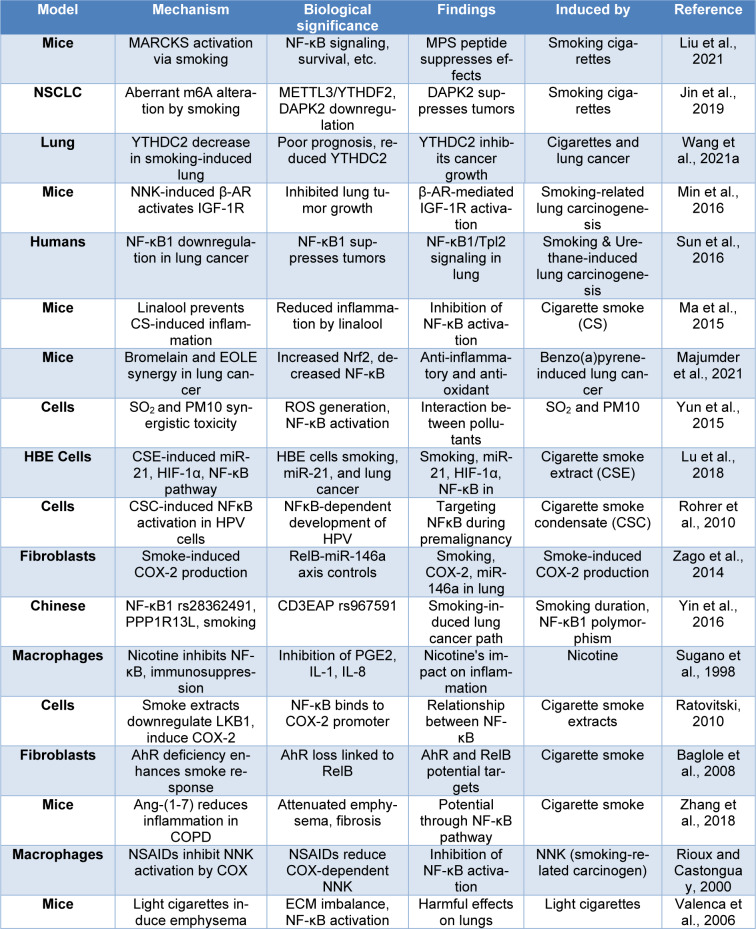
This table provides a concise summary of studies related to smoking-induced lung cancer, detailing various models, mechanisms, biological significance, key findings, and factors inducing changes in each study.

**Figure 1:**
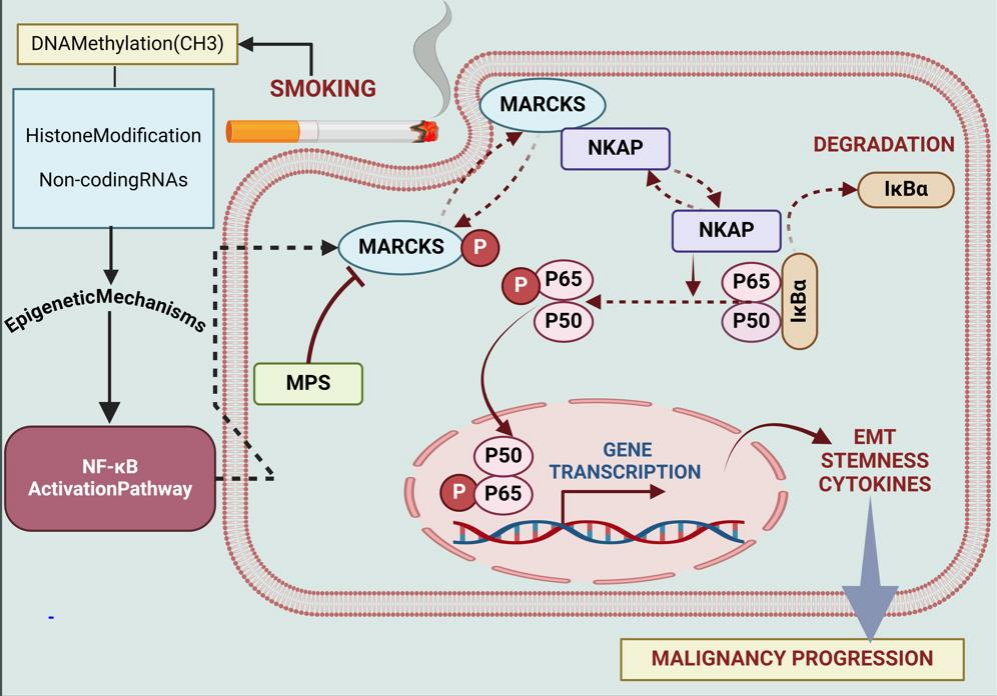
Graphical abstract
